# An Integrated Ecological Niche Modelling Framework for Risk Mapping of Peste des Petits Ruminants Virus Exposure in African Buffalo (*Syncerus caffer*) in the Greater Serengeti-Mara Ecosystem

**DOI:** 10.3390/pathogens12121423

**Published:** 2023-12-07

**Authors:** Laura Carrera-Faja, Chris Yesson, Bryony A. Jones, Camilla T. O. Benfield, Richard A. Kock

**Affiliations:** 1 Wildlife Conservation Medicine Research Group, Departament de Medicina i Cirurgia Animal, Universitat Autònoma de Barcelona, Edifici V, Travessera dels Turons, 08193 Cerdanyola del Vallès, Spain; 2 Institute of Zoology, Zoological Society of London, London NW1 4RY, UK; chris.yesson@ioz.ac.uk; 3 WOAH Collaborating Centre in Risk Analysis and Modelling, Department of Epidemiological Sciences, Animal and Plant Health Agency, Addlestone, Surrey KT15 3NB, UK; bryony.jones@apha.gov.uk; 4 Food and Agriculture Organization of the United Nations (FAO), Viale delle Terme di Caracalla, 00153 Rome, Italy; camilla.benfield@fao.org; 5 Department of Pathobiology and Population Sciences, Royal Veterinary College, University of London, London NW1 0TU, UK

**Keywords:** African buffalo, disease risk mapping, spatial epidemiology, maxent, peste des petits ruminants virus, PPR, PPRV, Serengeti-Mara

## Abstract

Peste des petits ruminants (PPR) is a highly contagious viral disease of small ruminants that threatens livelihoods and food security in developing countries and, in some cases, wild ungulate species conservation. The Greater Serengeti-Mara Ecosystem (GSME) encompasses one of the major wildlife populations of PPR virus (PPRV)-susceptible species left on earth, although no clinical disease has been reported so far. This study aimed to gain further knowledge about PPRV circulation in the GSME by identifying which factors predict PPRV seropositivity in African buffalo (*Syncerus caffer*). Following an ecological niche modeling framework to map host-pathogen distribution, two models of PPRV exposure and buffalo habitat suitability were performed using serological data and buffalo censuses. Western Maasai Mara National Reserve and Western Serengeti National Park were identified as high-risk areas for PPRV exposure in buffalo. Variables related to wildlife-livestock interaction contributed to the higher risk of PPRV seropositivity in buffalo, providing supportive evidence that buffalo acquire the virus through contact with infected livestock. These findings can guide the design of cost-effective PPRV surveillance using buffalo as a sentinel species at the identified high-risk locations. As more intensive studies have been carried out in Eastern GSME, this study highlights the need for investigating PPRV dynamics in Western GSME.

## 1. Introduction

Peste des petits ruminants (PPR) is a highly contagious disease of goats and sheep caused by an RNA single-stranded virus from the genus *Morbillivirus*, family *Paramyxoviridae*, closely related to the rinderpest virus [1]. PPR was first described in 1942 in Ivory Coast, although its emergence dates back to the beginning of the 20th century, and has since then expanded to Central and East Africa, the Middle East, and Central and South Asia [2]. It recently emerged in previously disease-free countries such as Mongolia, China, Kenya, Tanzania, Morocco, Tunisia [3], and Bulgaria [4]. Occurring in countries that contain 80% (approximately 1.7 billion head) of the world population of sheep and goats, PPR impacts the livelihoods, food security, and small ruminant trade of 300 million rural families, accounting for an estimated USD 2.1 billion economic loss per year [5]. In 2016, the World Organization for Animal Health (WOAH, founded as OIE) and the Food and Agriculture Organization of the United Nations (FAO) launched the Global Strategy for the Control and Eradication of PPR and the PPR Global Eradication Programme (PPR GEP) with the aim of eradicating this disease by 2030 [6].

One of the challenges to effectively eradicating PPR on a global scale is the need to understand the role of wildlife in PPR dynamics. Multiple wildlife species are susceptible to PPR virus (PPRV) infection, especially ungulates within the order Artiodactyla [3]. As PPRV is mainly transmitted via direct contact between susceptible and infected animals, important potential mechanisms for livestock-to-wildlife transmission include the sharing of water and grazing resources [7]. Introduction of PPRV from probable livestock origin has resulted in wildlife epidemics, such as in wild goats (*Capra aegagrus*) and goitered gazelles (*Gazella subgutturosa*) in Iran [8], Kurdistan [9], and a large-scale mortality event in the critically endangered Mongolian saiga (*Saiga tatrica mongolica*) population [10,11]. Thus, PPR poses an important threat to species conservation, and it is considered an emerging wildlife disease [12]. While major knowledge gaps still remain as to the epidemiological role of wildlife, it is important that countries, in particular those with intense livestock-wildlife interfaces, consider wildlife in their PPR National Strategic Plans and in their control and surveillance activities. PPRV is endemic in Kenya and Tanzania, the two countries that are associated with the Greater Serengeti Mara Ecosystem (GSME) [13]. Kenya was believed to be free from disease until 2007, although evidence suggests an earlier possible incursion in the north since antibodies in livestock were detected as early as 1995, but the virus did not establish epidemically until 2006 [14]. PPR in Tanzania was first reported in 2008 in Ngorongoro district (part of the GMSE), with 45.8% seroprevalence in small ruminants [15], and since then, seropositive cases and outbreaks have been detected in many parts of the country [16,17]. PPRV seroconversion has also been reported in cattle, indicating virus infection, although experimental studies indicate that cattle are dead-end hosts [18,19]. PPRV antibodies in free-ranging wildlife species from the GSME have been detected, which were considered to be a result of spillover events from domestic ruminants [17,20], although no clinical disease or mortality events have been reported to date. An earlier study of wildlife sampled before 2012 found no seroconversion, but evidence is limited due to biased sampling in wildlife-protected areas with low interaction with livestock [21].

In this ecosystem, the African buffalo (*Syncerus caffer*) has a relatively high PPRV seroprevalence among sampled wildlife species [17]. It is a philopatric species that tends to remain in the same area [22], and within the GSME, it is highly restricted to the Serengeti National Park (SNP) and Maasai Mara National Reserve (MMNR). Thus, the boundaries of these protected areas provide a linear interface with livestock. Since buffalos are highly water-dependent compared to other species, they interact intensively with livestock by sharing resources such as pastures and water, especially in drought conditions. Moreover, buffalo may be dead-end hosts for PPRV, as is the case for cattle [17,20]. In such instances, PPRV transmission within buffalo populations or from buffalo to livestock would be unlikely. Therefore, the African buffalo could be a suitable indicator of PPRV circulation and a potentially good sentinel at this interface.

Spatial analysis in animal and public health has the aim of describing existing geographical patterns of disease risk, analysing the mechanisms of disease occurrence, and predicting the outcomes in the future or in different areas [23]. Different methods for temporal or spatial models have been used in the field of infectious diseases. Depending on the type of input, models can be data-driven (based on observational data) or knowledge-driven (based on available evidence and knowledge) [24].

Ecological Niche Modelling (ENM) is a data-driven approach widely used in conservation biology, ecology, and evolution. The ENM approach uses computer algorithms to estimate the similarity of the conditions in any geographical area to the conditions at the locations of known occurrences of a phenomenon. The most common application is to predict species distributions across landscapes using occurrence data (georeferenced points in which the species have been detected) and GIS layers of environmental variables (e.g., rainfall, temperature, elevation) [25]. ENM has been applied to disease transmission systems with the aim of exploring how environmental conditions influence the occurrence of disease or vectors, understanding disease ecology, identifying unknown vectors or hosts, and predicting potential areas of disease occurrence or changes in disease distribution under climate change scenarios [26]. For example, this approach has been used to model avian influenza at the wildlife–livestock interface [27] and to predict the presence of *Culex pipens*, the vector of West Nile and Rift Valley Fever [28,29]. However, directly-transmitted diseases and pathogens less dependent on environmental persistence are more difficult to model using ENM since this approach cannot account for fine-scale factors (e.g., age, immune status) that might be required to reliably reconstruct disease presence. Nevertheless, ENM can be useful to map pathogen occurrences of diseases in which reservoirs or multiple species are involved in the transmission cycle [30].

Spatial modelling of PPRV in livestock has already been applied in East Africa [31], China [32], and worldwide [33], using different approaches—GIS-based multicriteria analysis (knowledge-driven model) and ENM (data-driven model). To our knowledge, spatial modelling of PPRV focusing on wildlife has not yet been attempted. One of the reasons is the difficulty in obtaining appropriate, non-biased data for disease occurrence with a robust sample size in wildlife. In the present study, data obtained in a cross-sectional serological survey in African buffalo, carried out during 2018–2019, were used to develop a spatial model for PPRV in wildlife using ENM. MaxEnt (short for ‘maximum entropy’)—a machine-learning algorithm that produces predictions of habitat suitability [34]—was the ENM algorithm chosen because it works well with small sample sizes, does not rely on absence data, can model non-linear variables, considers sampling bias, and does not assume that observations are independent [24].

Thus, our objective was to explore which environmental variables predicted habitat suitability—used as a synonym for probability of occurrence—for both PPRV antibody-positive buffalo herds and the presence of buffalo across the GSME to detect high-risk areas of PPRV-seropositive buffalo herds. Through this integrative ENM framework, this study aimed to provide further understanding of PPRV risk factors in wildlife and PPRV transmission dynamics and help stakeholders determine cost-effective PPR surveillance and control measures. We hypothesise that areas with higher livestock densities and closer proximity to the border of protected areas have a higher risk of buffalo PPRV exposure [35].

## 2. Materials and Methods

### 2.1. Study Area

**Figure 1:**
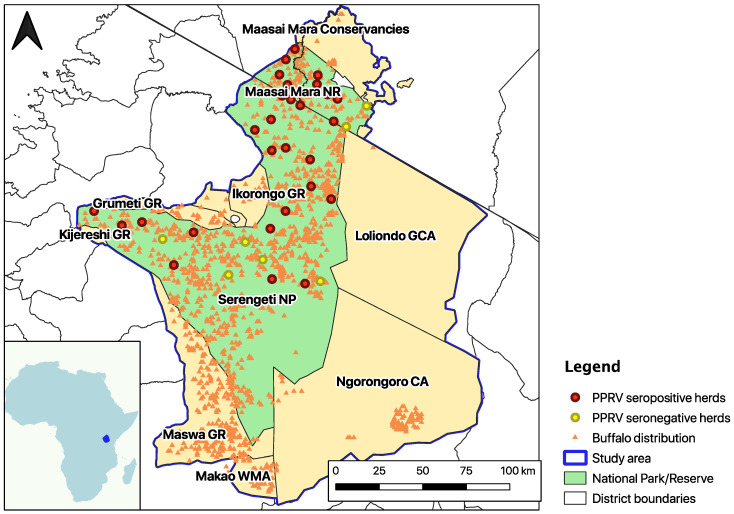
Map of the Greater Serengeti-Mara ecosystem (GSME), outlined in purple. Red dots indicate the sampling sites with at least one PPRV N cELISA positive buffalo (positive percentage inhibition (PI) < 60), yellow dots indicate the sites with no PPRV cELISA positive buffalo herds, and orange triangles show the sightings of one or more buffalos during the 2014 dry and wet season censuses [40,41]. The area in green comprises Serengeti National Park (NP) and Maasai Mara National Reserve (NR), the protected areas where livestock is not allowed. The pale-yellow regions are Wildlife Management Area (WMA), Game Controlled Area (GCA), Conservation Area (CA), and Game Reserve (GR).

The Greater Serengeti-Mara ecosystem (GSME) (Figure 1) encompasses over 35,000 km^2^ of wildlife-dominated land in Northern Tanzania and Southwestern Kenya. It includes grassland, Acacia savanna, riverine, and woodland vegetation. Temperatures oscillate between 16 and 27 °C, and the mean annual rainfall is 800–1000 mm. This ecoregion is famous for its biodiversity and is home to increasing human populations adjacent to the GSME protected areas [36], such as Maasai and other agro-pastoralist communities and their livestock [37]. It hosts a great density and species diversity of wild herbivores, including African buffalos, zebras (*Equus burchelli*), Grant’s gazelles (*Nanger granti*), Thompson’s gazelles (*Gazella thomsoni*), over one million wildebeests (*Connochaetes taurinus*), and many other antelopes and wild suids [38]. Protected areas in the GSME (i.e., Serengeti National Park and Maasai Mara National Reserve) provide many ecosystem services, but human impacts such as landscape degradation at their edges compromise ecosystem health [39].

### 2.2. Data Sources and Preparation

#### 2.2.1. PPRV Seropositive Buffalos in the GSME

Presence data on PPRV seropositive buffalos were obtained from a cross-sectional study carried out in 2018–2019 with the aim of determining the PPRV seroprevalence in two resident wild species: African buffalo and Grant’s gazelle. It consisted of a two-stage survey design with herds as the first stage and animals as the second stage, sampling 29 randomly selected sites within the species range and 5 animals per site, plus a more intensive sample of all six buffalo herds in the Mara Triangle (Western part of the Maasai Mara National Reserve) and eight animals per herd [17]. In this study, only the results of the buffalo survey were used (Figure 1). Jones et al. (2021) [17] used the ID Screen PPR Competition ELISA (cELISA) for the detection of anti-PPRV nucleoprotein (N) antibodies (IDvet, Gravels, France, https://www.id-vet.com/produit/id-screen-ppr-competition/(accessed on 19 August 2022)) in a total of 35 buffalo herds, considering a herd positive if at least one sampled individual gave a percentage inhibition (PI) value < 50 in the cELISA, as per manufacturer recommendations. Since this test is only validated for domestic animals and not for wildlife, Jones et al. (2021) [17] also explored a second cELISA interpretation in which all <60 PI values were considered positive, thus increasing sensitivity while decreasing specificity. The present study used the results of the second interpretation, considering 28 georeferenced occurrence points (i.e., PPRV antibody-positive buffalo herds). This was conducted to increase the number of occurrence points and enhance the predictive performance of the model. The model was also re-run using the <50 PI cut-off values, which corresponded to 16 observations, in order to explore the effect of different cELISA interpretations on the results (Supplementary Materials, Figure S1).

#### 2.2.2. Buffalo Presence Points in the GSME

Presence data on buffalo sightings (*n* = 1506) were obtained from the 2014 Aerial census by the Tanzania Wildlife Research Institute [40] and the 2014 Aerial census by the Kenya Wildlife Service (KWS) during the dry and wet seasons [41] (Figure 1).

#### 2.2.3. Selection of Predictor Variables of PPRV Antibody Positive Buffalo Herds

Variables hypothesised to affect exposure to PPRV infection in wild and domestic species and buffalo presence were identified in the literature [21,31,32,33,35,42,43], and those considered for this study are presented in Table 1. Sheep and goat density were combined into a single layer using the QGIS (v. 3.16.4) ‘raster calculator’ tool, and the two variables describing proximity to the features ‘border of protected areas’ and ‘bomas’ were created using the QGIS Euclidean distance tool. All variables were clipped to the extent of the GSME (Figure 1) with a 0.002 degree resolution (~220 m).

#### 2.2.4. Collinearity Analysis

In order to avoid collinearity and model overfitting, the Variance Inflation Factors (VIFs) of the 25 variables were tested using the ‘sdm’ package in Rstudio 1.4 1103. VIF quantifies the severity of multicollinearity that variables present based on the square of the multiple correlation coefficient (R2), and those with VIF > 10 were excluded [48]. The twelve remaining variables were included in the models: proximity to bomas (km), proximity to the border of protected areas (km), cattle density (head/km^2^), sheep and goat density (head/km^2^), Normalized Difference Vegetation Index (NDVI) maxima, International Union of Conservation of Nature (IUCN) habitat type, temperature seasonality (bio4, coefficient of variation, °C/100), temperature annual range (bio7, °C), mean temperature of the wettest period (bio8, °C), precipitation of the wettest period (bio13, kg/m^2^), precipitation of the driest period (bio14, kg/m^2^), and precipitation of the warmest quarter (bio18, kg/m^2^).

### 2.3. Modelling Habitat Suitability for PPRV and Buffalo Occurrence

MaxEnt software version 3.4.4 [49] was used for the ecological modelling of the habitat suitability of PPRV antibody-positive buffalo herds (positive herd model) and buffalo presence (buffalo model). Our presence data on PPRV antibody in buffalo was considered to be spatially biased because, even though sampling sites were randomly selected, some were excluded due to difficult accessibility and replaced by other randomly selected nearby sites, so there was bias towards more accessible areas. Moreover, sampling was only performed within Serengeti National Park (NP) and Maasai Mara National Reserve (NR), and we assumed that all the buffalo habitat ranges in GSME could be suitable for buffalo PPRV exposure. A bias file reflecting sampling intensity across the study area was created to restrict background points [50]. A Gaussian kernel density map of the 35 sampling sites (both positive and negative for PPRV antibodies) was created using the ‘kde2d’ function in Rstudio 1.4 1103, with normal reference bandwidth values through Silverman’s rule of thumb, assuming normal distribution of density [51].

The ENMevaluate function from the ‘ENMeval’ R package was used to rigorously calibrate and evaluate MaxEnt settings for best model performance [52]. The model with the lowest sample-size corrected Akaike Information Criterion (AICc) value (i.e., DAICc = 0) was selected. A random k-fold cross-validation suggested for small samples in which the number of bins (k) is the number of localities (k = 28) was used to estimate the performance of the positive herd model [53]. The model was calibrated with 280 randomly generated background points from the bias file (=10× presence points). For the buffalo model, we used a random k-fold cross-validation (k = 10), which provides an optimal balance between computational cost and low bias in the estimate of the model performance, and 15,060 background points (=10× presence points). Tuning for the positive herd model resulted in a regularisation multiplier of 1.5 and the use of linear and quadratic features, while Buffalo used a regularisation multiplier of 1 and linear, quadratic, hinge, threshold, and product features.

Response curves were created, and the contribution of each variable to the model was tested through a jackknife procedure [34,54]. Both the positive herd model and buffalo model were run at a convergence threshold of 0.00001, 500 iterations, and MaxEnt complemental log–log (cloglog) output with all other parameters left at their defaults.

## 3. Results

### 3.1. Variable Contribution, Model Performance, and Response Curves

#### 3.1.1. Buffalo Model

The predictive performance was good, with an average test AUC of 0.858 and a standard deviation of 0.010. NDVI maxima, proximity to borders, and temperature annual range (bio 7) accounted for 25.6%, 23.7%, and 18.8% of model variation, respectively (Table 2).

**Figure 2:**
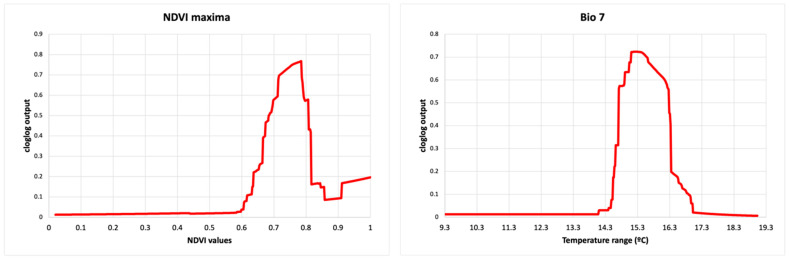
Response curves for the Buffalo model (cloglog output) in relation to the maximum Normalised Difference Vegetation Index (NDVI maxima) (**left**) and temperature annual range (Bio 7) (**right**). The red line represents the mean response of the 10 replicates.

**Figure 3:**
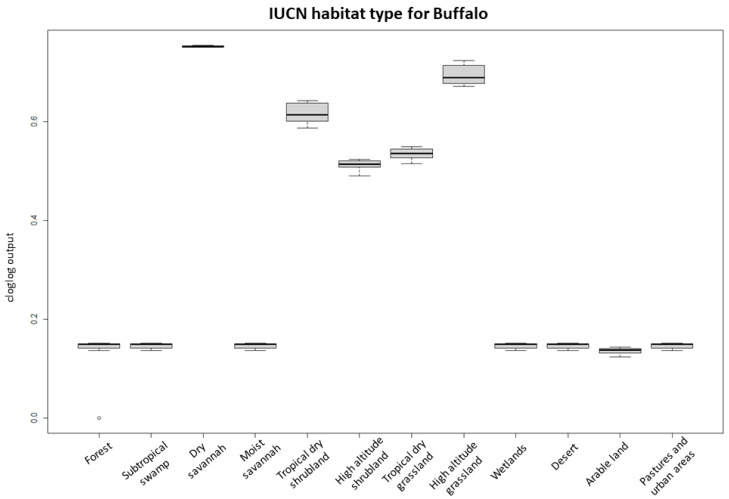
Probability of buffalo occurrence in relation to the IUCN habitat classification. The boxplots represent the mean (black line within the box) of the 10 replicates for the probability scores of the different habitat types. The upper and lower box boundaries correspond to the 1st and 3th quartiles and the upper and lower error lines represent 1.5 times the interquartile range, with circles as outliers.

The highest suitability score for buffalo occurrence showed a peak between 0.7 and 0.8 of NDVI maxima, between 15 and 16 °C of the annual temperature range (bio7) (Figure 2), at ~4.4–5.5 km from bomas and with low sheep and goat density. Habitat suitability for buffalo also had the highest score in areas close to the border of protected areas and dry savannah, high-altitude dry grassland, and tropical dry shrubland IUCN habitat, while arable and pasture land and desert had the lowest probabilities of buffalo occurrence (Figure 3).

#### 3.1.2. PPRV Antibody Positive Buffalo Herd Model

**Figure 4:**
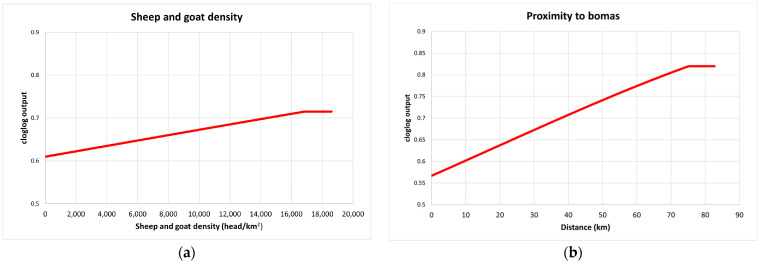
Response curves for the PPRV antibody-positive model (cloglog output) in relation to sheep and goat density (head/km^2^) (**a**) and proximity to bomas (**b**). The red line represents the mean response of the 28 replicates.

**Figure 5:**
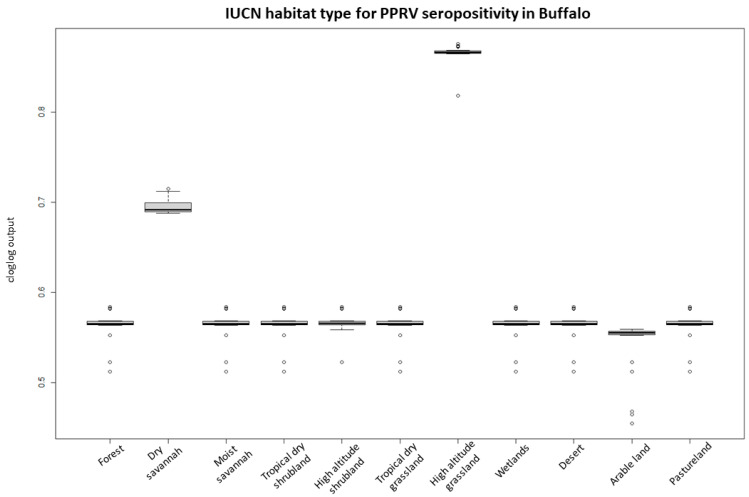
Probability of PPRV antibody-positive buffalo herds occurrence (cloglog output) in relation to the IUCN habitat classification. The boxplots represent the mean (black line within the box) of the 28 replicates for the probability scores of the different habitat types. The upper and lower box boundaries correspond to the 1st and 3th quartiles and the upper and lower error lines represent 1.5 times the interquartile range, with circles as outliers.

For the PPRV antibody-positive buffalo herd model, the predictive performance was considered good, with an average test AUC of 0.797 for the 28 replicates and a standard deviation of 0.193. IUCN habitat type was the most important variable for the model, accounting for 71% of variation and being the most predictive single parameter (Table 2). Response graphs showed that habitat suitability for PPRV antibody-positive herds (represented as cloglog output) linearly increased as sheep and goat density increased and as the distance (km) to the nearest boma increased (Figure 4). IUCN habitats with the highest habitat suitability for PPRV were high-altitude grasslands and dry savannah, whereas arable land had the lowest suitability (Figure 5).

### 3.2. Habitat Suitability Maps

#### 3.2.1. Buffalo Suitability Map

**Figure 6:**
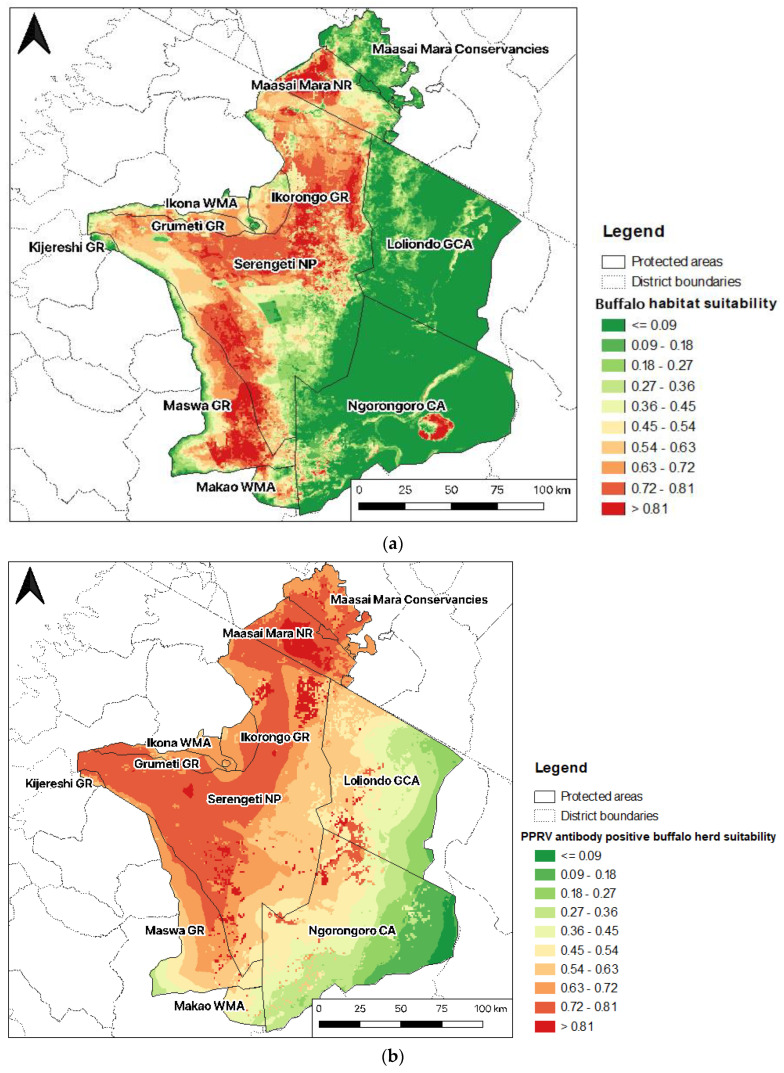
Predicted distribution of buffalo (**a**) and PPRV antibody-positive buffalo herds (**b**) in the Greater Serengeti-Mara ecosystem.

The buffalo probability of occurrence was very high in the Mara Triangle in the western part of the Maasai Mara NR (>0.81), representing almost 50% of the MMNR. Almost half of the area of Serengeti NP (48%) had high suitability scores (>0.72), mainly in the north, centre, and southwest, bordering Maswa GR, which also had a very high habitat suitability score. Loliondo GCA and Ngorongoro CA were not suitable for buffalo occurrence, except in the area of the Ngorongoro crater (Figure 6a).

#### 3.2.2. PPRV Antibody Positive Buffalo Herd Suitability Map

Maasai Mara NR, along with the northeastern part of Serengeti NP, presented the highest habitat suitability scores for PPRV antibody-positive buffalo herds (>0.81) (Figure 6a). The western part of Serengeti National Park was also highly suitable for PPRV seropositivity in buffalo (0.72–0.81), especially areas bordering Ikorongo, Grumeti, Kijreshi, and Maswa Game Reserves (GR). There were also patches of higher suitability in adjacent areas of the northwestern Ngorongoro Conservation Area (CA) and Loliondo Game Controlled Area (CA). In contrast, the eastern parts of the GSME in Loliondo GCA and Ngorongoro CA had very low habitat suitability (Figure 6b).

### 3.3. PPRV Antibody Positive Herd Suitability Considering the <50 PI Cut-Off Value

When the <50 PI cut-off was used to identify PPRV-antibody positive herds, the predictive performance of the model was low (AUC = 0.610), and the variables that contributed the most were proximity to borders (62.1%)—the closer to the borders, the higher the habitat suitability—, IUCN habitat (19.2%), and NDVI maxima (6.2%) (Supplementary Materials, Table S1). The PPRV antibody herd suitability map was spatially homogeneous within the Serengeti NP and Maasai Mara NP with scores ranging 0.63–0.72, apart from lower suitability in Central Serengeti NP, Eastern Loliondo GCA, and Ngorongoro CA (Supplementary Materials, Figure S2).

## 4. Discussion

This study is the first to use an ENM approach to examine PPRV risk, which highlights areas for wildlife disease surveillance [55]. Prior to this study, ENMs for PPRV occurrence had only been performed adopting ‘black-box’ approaches by modelling the overall PPRV distribution and likely oversimplifying biotic interactions involved in transmission, neglecting ecological complexity [32,33].

It is worth highlighting that the main objective of this study was to spatially determine the risk of PPRV exposure in buffalo herds and not the overall PPRV risk in all susceptible species in the GSME. Since only data on PPRV seropositive buffalos were examined, the positive herd model (Figure 6b) reflects habitat suitability for PPRV antibody-positive buffalo herds and should not be interpreted more generally or extrapolated to other host species. Despite being dependent, both positive herd and buffalo ENMs presented different outputs, and characterising habitat suitability for both host and pathogen by overlapping their distributions [56] allowed a more comprehensive characterization of the spatial risk of PPRV exposure in buffalo and a preliminary PPR risk assessment for buffalo that indicated areas to be targeted for enhanced surveillance.

Our results showed that areas within and near the borders of Serengeti NP and Maasai Mara NR, which comprise the buffalo–livestock interface, were at high risk of PPRV antibody positivity in buffalo. This provides further evidence that African buffalos are likely to be spillover hosts, and virus infection occurs predominantly from infected livestock that are present in areas adjacent to protected areas rather than from other wildlife species or buffalos [35,57]. The low PPRV risk in zones immediately adjacent to bomas supported the hypothesis that PPRV transmission is more likely to occur in pastures or water sources rather than closer to human settlements with high livestock density, which are avoided by buffalos [58].

Higher PPRV risk was expected in areas neighbouring Loliondo GCA (i.e., the northeastern part of Serengeti NP) due to regular outbreak reports in sheep and goats [17,20,59,60,61,62], but areas in the central east of SNP had low habitat suitability for buffalo and thus a lower probability of buffalo exposure to PPRV. In that sense, NDVI, which is an indicator of vegetation cover/density and also related to rainfall [63], was the variable that mostly explained the potential distribution of buffalos within the GSME, reflecting the fact that this species is restricted to areas with higher nutritional pastures and dependent on water sources, especially during the dry season [22]. IUCN habitat type, proximity to borders, and annual temperature range also made important contributions to this model and displayed similar spatial patterns, since the most suitable habitat types for buffalo (i.e., dry savannah, grasslands, and shrublands) encompassed both optimal NDVI and temperature ranges for habitat suitability, whereas areas near borders tended to have lower vegetation cover because of livestock grazing [39] and consequently lower habitat suitability for buffalo. In summary, in some areas where PPRV risk was expected to be high due to the wildlife–livestock interface and the occurrence of PPRV disease in sheep and goats, the risk is low because the habitat is less suitable for buffalo.

In the Western Serengeti NP, agro-pastoralist herds illegally enter protected areas for short night grazing trips, especially during the dry season when there is a shortage of grazing [39], which likely favours the transmission of PPRV considering that night and early morning climatic conditions could allow the virus to survive for short periods in the environment (shade and higher humidity, e.g., dew on grass) [64]. Moreover, in the Northeastern Serengeti NP and Maasai Mara NR, Maasai pastoralists have been reducing the number of cattle kept and increasing the number of domestic small ruminants as a consequence of recurrent droughts and land-use changes, which is evidenced by an increase in sheep and goat density in the Maasai Mara ecosystem [65]. Apart from competing with wildlife for grazing and potentially changing the local ecosystem [66], the increasing small ruminant density in other areas of the GSME, such as areas surrounding the Serengeti NP, and its impact on potential PPRV transmission to buffalo require further investigation.

Even though IUCN habitat is influenced by weather conditions and is a major contributor to the PPRV antibody-positive buffalo herd model, the rest of the climatic variables only made a small contribution to it, probably because the study area is a semi-arid area and climatic variables showed high homogeneity. For instance, while a PPR ENM study in China found that PPR habitat suitability was highest when the precipitation of the driest month (bio14) was 20 mm [32], precipitation values of the driest month in our study area only ranged from 0 to 4 mm. Moreover, despite playing a possible role in PPRV short-term survival in the environment, which might favour virus transmission, climatic variables were also thought to increase the severity of PPR disease in wildlife in Mongolia [10]. In the GSME, there has been no apparent clinical disease in PPRV susceptible wild species. The present study therefore only assessed the risk of PPRV seropositivity as an indicator of virus spillover and could not explore drivers of disease expression.

As PPRV sampling was focused on the northern, central and western parts of Serengeti NP and the Maasai Mara NR, where buffalo herds were found during the sampling campaigns, the model could only make confident predictions in these regions [67,68]. Therefore, in order to increase this model’s reliability for the whole GSME, further studies should focus on model ground-truthing by comparing PPRV seroprevalence in buffalo in areas with different habitat suitability scores for PPRV antibody-positive herds. The cross-sectional serological survey of buffalo did not include herds in the Ngorongoro crater because it was not possible to obtain permission to dart a large number of buffalo in that area. There have been several PPR outbreaks in livestock in areas nearby [20,59], so our study may have failed to predict a high PPRV risk there. Similarly, no buffalos were sampled in Maswa GR.

Even if the PPRV antibody-positive buffalo herd model provided reliable evaluation values (i.e., good predictive performance), relevant environmental variables may have been missed due to the lack of availability of geo-referenced data, such as spatial information on resources shared by wildlife and livestock that increase the probability of interspecies pathogen transmission (e.g., salt licks, water points, and riverine systems [69]. Moreover, in order to assess the possibility of PPRV transmission involving multiple host species, as occurred in Iran and Mongolia [8,10], biotic interactions such as the density of other PPR-susceptible species and seroprevalence in other species should be explored. This could include migratory antelopes such as wildebeest or other hosts such as Thompson’s gazelle and impala.

This study was constrained by the small sample size since it was initially designed to determine seroprevalence and not for risk factor analysis [17]. However, a larger sample size would not have been possible due to the logistical, economic, and welfare constraints of capturing and sampling large numbers of wild animals in a remote area. The presence of antibody-positive animals in a herd only indicates previous infection with PPRV and not infection at the time of sampling; therefore, the exact location when PPRV infection occurred is unknown, although buffalo are a philopatric species and do not move long distances, only a few kilometres [22]. Moreover, the duration of antibody persistence in this species is not known. This study used the results of the IDvet cELISA test to identify African buffalo herds with one or more PPRV antibody-positive animals. A recent study has shown that this test has relatively low sensitivity (37.5%) when compared to the VNT but relatively high specificity (88.6%) when using the manufacturer’s recommended cutoff (<50) [70]. Using the higher cutoff (<60) leads to the same low sensitivity but lowers the specificity, leading to more false positives. If the VNT is assumed to be highly sensitive and specific, then in our study some true positive herds could have been misclassified as negative herds, but most of the positive herds were true positives. Given the uncertainty around the interpretation of the cELISA results in this species, the results of this study should be considered a demonstration of the application of this method for this species and pathogen rather than an accurate representation of the situation in this ecosystem. Since the current study was based on available datasets, models could be re-run once updated datasets become available and confirmatory tests such as VNT are performed. This study also explored the <50 PI cut-off interpretation as a sensitivity analysis. We observed that re-running the model using this interpretation did not substantially alter the overall output, but it considerably reduced both predictive performance and spatial heterogeneity in habitat distribution scores (Supplementary Materials, Figure S2), questioning its reliability.

Similar to rinderpest virus (RPV) eradication, in which wildlife species did not act as reservoirs and were valuable sentinels for infection in livestock [71,72], monitoring buffalo seroprevalence is likely to be useful during the later stages of PPR eradication in countries with large buffalo populations. However, such spatial models focusing on wildlife were not needed to guide policy for the successful eradication of RPV in Eastern Africa. This was mainly because wildlife acted as a strong indicator of rinderpest virus circulation in contiguous cattle populations due to the occurrence of severe clinical disease in buffalo during epidemics and the high seroprevalence in the buffalo that survived the disease [71]. The seroprevalence in buffalo and other wild susceptible species was shown to decline rapidly once RPV was no longer circulating in cattle, confirming that wildlife was not able to maintain the virus and act as a reservoir. Nevertheless, spatial modelling is a useful tool to identify hotspots for sentinel surveillance in wild species, especially where overall seroprevalence in wild species is low, such as with PPRV. However, it relies on a representative sample from the population, which is a resource-intensive exercise.

In terms of disease control and wildlife management, parts of the GSME have adopted the multiple land-use system, in which traditional pastoralists and farmers share the natural resources with wildlife in protected areas. Although this is sustainable and beneficial for ecosystem health and developing economies in the long term [73], these complex transboundary ecosystems require multidisciplinary approaches for disease surveillance and control to take into consideration the political, cultural, and economic context [74]. Hence, PPR eradication will require a holistic international cooperative approach, and the emerging field of spatial modelling could make an important contribution.

## Figures and Tables

**Table 1:** Variables associated with PPRV circulation and buffalo presence in the GSME for which spatial data were available, their hypothesised effects, and source of geographical data.

Environmental Variables	Hypothesis	Data Source
Sheep and Goat Density	Small ruminant density contributes to PPRV habitat suitability in coarse-scale models [33]. As livestock is thought to transmit PPRV to wildlife [20], sheep and goat density is an important factor in the Greater Serengeti-Mara Ecosystem (GSME).	Gridded Livestock of the World 3 database [44], downloaded from FAO GeoNetwork (http://www.fao.org/livestock-systems/global-distributions/en/ (accessed on 23 June 2021)) at a 5 arc-minute resolution (~10 km).
Cattle Density	Cattle infection is an indicator of PPRV circulation in sheep and goats and may be a good sentinel for PPRV at the wildlife-livestock interface [45]. In general, cattle are more likely to enter protected areas than goats and sheep [35].	Gridded Livestock of the World 3 database [44], downloaded from FAO GeoNetwork (http://www.fao.org/livestock-systems/global-distributions/en/ (accessed on 23 June 2021)) at a 5 arc-minute resolution (~10 km).
IUCN habitat types	Buffalo distribution depends on the habitat type, and consequently, higher probabilities of PPRV occurrence in buffalo might be restricted to certain habitat types.	International Union of Conservation of Nature (IUCN) habitat classification scheme [46].
Temperature and rainfall	Both temperature and rainfall contribute to PPRV habitat suitability in coarse-scale ENMs, but it is unknown in African buffalo [32,33,43]. Since important mechanisms for PPRV transmission between wildlife and livestock are the sharing of grazing and water resources [17], climatic variables might play a role in PPRV short-term persistence in the environment and shared sites [43].	19 bioclimatic variables related to temperature and rainfall. Worldclim website (www.worldclim.org, (accessed on 23 June 2021)) at a 30 arc-second resolution (~1 km) [47].
Proximity to the border of protected areas	The borders of protected areas where livestock is not allowed (Maasai Mara National Reserve and Serengeti National Park) are mainly open, and both wildlife and livestock can freely cross them, making them a potential area for high-risk PPRV transmission.	Serengeti-Mara Ecosystem Protected Areas. Serengeti GIS and Data: https://serengetidata.weebly.com/data.html (accessed on 23 June 2021) [39] at a 0.002 degrees resolution (~220 m).
Proximity to bomas	Areas surrounding bomas where livestock are enclosed at night tend to be degraded, and wildlife does not approach them. However, the livestock from bomas in areas bordering Serengeti National Park and Maasai Mara National Reserve may be illegally taken inside the restricted areas for grazing, increasing the probability of PPRV livestock-wildlife transmission.	Boma Distribution. Serengeti GIS and Data https://serengetidata.weebly.com/data.html (accessed on 23 June 2021) [39] 0.002 degree resolution (~220 m).
NDVI maxima	The Normalised Difference Vegetation Index (NDVI) is an indicator of green vegetation. Areas with greener and richer pastures might gather more wildlife species and livestock together, and the probability of PPRV transmission might be higher.	Serengeti GIS and Data: https://serengetidata.weebly.com/data.html [39], during 2011–2016, at 5.6 × 10^−5^ degrees resolution (~6.24 m)

**Table 2:** Estimates of relative contributions of the individual predictor variables to the PPRV (left) and buffalo (right) MaxEnt models, assessed through the jackknife of regularized training gain in each iteration by running the models in isolation and comparing them to the training gain of the complete models.

PPRV Seropositivity in Buffalo		Buffalo	
Variable	Percent Contribution	Variable	Percent Contribution
IUCN habitat	71.2	NDVI maxima	25.6
Sheep and goat density	15.4	Proximity to borders	23.7
Proximity to Bomas	5.7	Bio 7	18.8
Bio 4	4	IUCN habitat	8.6
Proximity to borders	2.6	Proximity to bomas	6.4
Bio 13	1	Sheep and goat density	5.2
Cattle density	0.1	Bio 8	4.8
Bio 7	0.0	Bio 13	2.3
Bio 18	0.0	Bio 4	2.0
Bio 8	0.0	Bio14	1.3
Bio 14	0.0	Cattle density	0.7
NDVI maxima	0.0	Bio 18	0.6

## Data Availability

The original contributions presented in this study are included in the article; further inquiries can be directed to the corresponding authors.

## Supplementary Materials

The following supporting information can be downloaded at: https://www.mdpi.com/article/10.3390/pathogens12121423/s1, Figure S1: Map of the Greater Serengeti-Mara ecosystem (GSME), highlighted in purple. Red dots indicate the sampling sites with at least one PPRV N cELISA-positive buffalo (positive percentage inhibition (PI) < 50), yellow dots indicate the PPRV-negative sites, and orange triangles show the sightings of one or more buffalos. Figure S2: Predicted distribution of PPRV in the Greater Serengeti-Mara ecosystem, considering PPRV N cELISA-positive buffalos as PI < 50; Table S1: Estimates of relative contributions of the individual predictor variables to the PPRV MaxEnt model considering PPRV N cELISA positive buffalos when percentage inhibition (PI) < 50, assessed through the jackknife of regularized training gain in each iteration by running the models in isolation and comparing them to the training gain of the complete models.
