# Supplementary material for: An Integrated Ecological Niche Modelling Framework for Risk Mapping of Peste des Petits Ruminants Virus Exposure in African Buffalo (Syncerus caffer) in the Greater Serengeti-Mara Ecosystem
Source: Pathogens. 2023 Dec 7;12(12):1423. doi: 10.3390/pathogens12121423 (PMC10747384; doi:10.3390/pathogens12121423)
Supplement: Supplementary file 1 [file pathogens-12-01423-s001.zip › Supplementary material - figure S1.pdf]

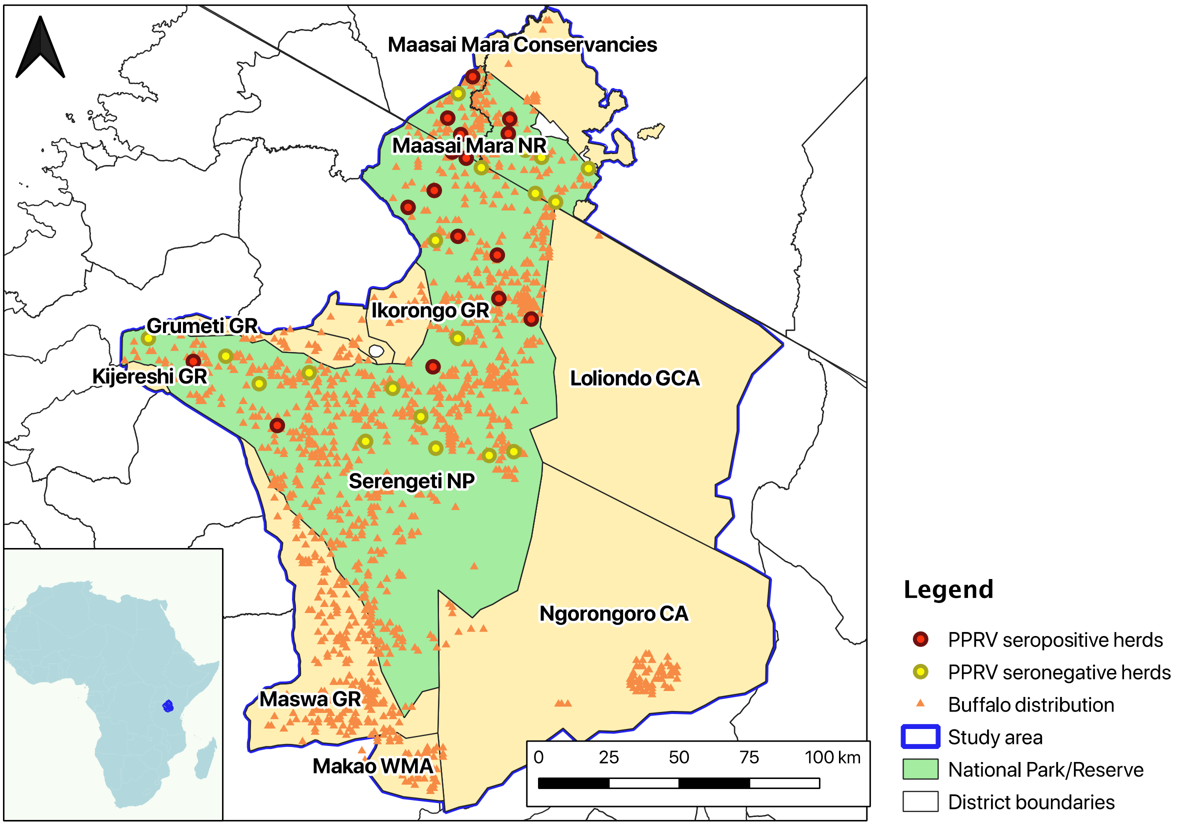

Figure S1. Map of the Greater Serengeti-Mara ecosystem (GSME), highlighted in purple. Red dots indicate the sampling sites with at least one PPRV N cELISA positive buffalo (positive percentage inhibition (PI) < 50), yellow dots indicate the PPRV-negative sites and orange triangles show the sightings of one or more buffalos.
