# Supplementary material for: An Integrated Ecological Niche Modelling Framework for Risk Mapping of Peste des Petits Ruminants Virus Exposure in African Buffalo (Syncerus caffer) in the Greater Serengeti-Mara Ecosystem
Source: Pathogens. 2023 Dec 7;12(12):1423. doi: 10.3390/pathogens12121423 (PMC10747384; doi:10.3390/pathogens12121423)
Supplement: Supplementary file 1 [file pathogens-12-01423-s001.zip › Supplementary material - figure S2.pdf]

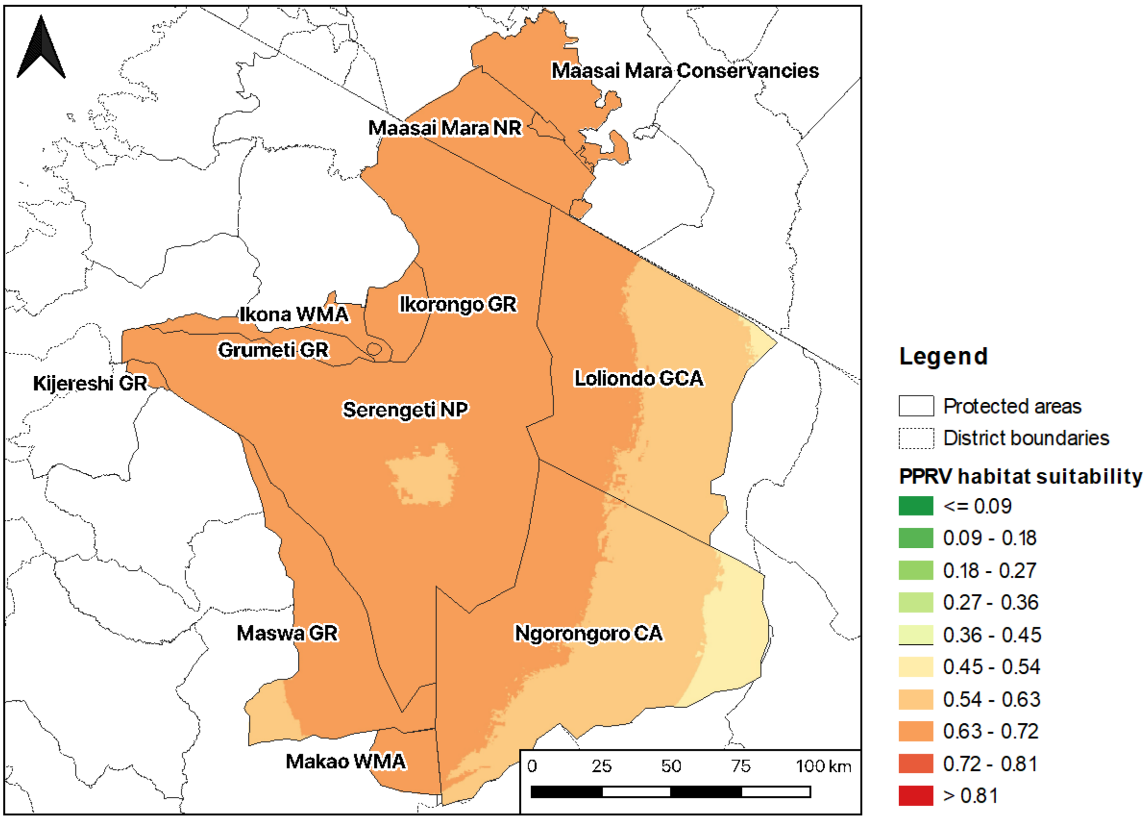

Figure S2. Predicted distribution of PPRV in the Greater Serengeti-Mara ecosystem, considering PPRV N cELISA positive buffalos as PI < 50.
