# Supplementary material for: An Integrated Ecological Niche Modelling Framework for Risk Mapping of Peste des Petits Ruminants Virus Exposure in African Buffalo (Syncerus caffer) in the Greater Serengeti-Mara Ecosystem
Source: Pathogens. 2023 Dec 7;12(12):1423. doi: 10.3390/pathogens12121423 (PMC10747384; doi:10.3390/pathogens12121423)
Supplement: Supplementary file 1 [file pathogens-12-01423-s001.zip › Supplementary material -Table S1.pdf]

Table S1. Estimates of relative contributions of the individual predictor variables to the PPRV MaxEnt model considering PPRV N cELISA positive buffalos when percentage inhibition (PI) < 50, assessed through the jackknife of regularized training gain in each iteration by running the models in isolation and comparing them to the training gain of the complete models.

**PPRV in buffalo**	
**Variable**	**Percent Contribution**
Proximity to borders	62.1
IUCN habitat	19.2
NDVI maxima	6.2
Mean temperature of the wettest period (Bio 8)	0.0
Temperature annual range (Bio 7)	0.0
Temperature seasonality (Bio 4)	0.0
Precipitation of the warmest quarter (Bio 18)	0.0
Precipitation of the driest period (Bio 14)	0.0
Precipitation of the wettest period (Bio 13)	0.0
Sheep and goat density	0.0
Proximity to bomas	0.0
Cattle density	0.0
